# A Computational Method for Optimizing Experimental Environments for* Phellinus igniarius* via Genetic Algorithm and BP Neural Network

**DOI:** 10.1155/2016/4374603

**Published:** 2016-08-09

**Authors:** Zhongwei Li, Beibei Sun, Yuezhen Xin, Xun Wang, Hu Zhu

**Affiliations:** ^1^College of Computer and Communication Engineering, China University of Petroleum, Qingdao, Shandong 266580, China; ^2^Center for Bioengineering and Biotechnology, China University of Petroleum, Qingdao, Shandong 266580, China

## Abstract

Flavones, the secondary metabolites of* Phellinus igniarius* fungus, have the properties of antioxidation and anticancer. Because of the great medicinal value, there are large demands on flavones for medical use and research. Flavones abstracted from natural* Phellinus* can not meet the medical and research need, since* Phellinus* in the natural environment is very rare and is hard to be cultivated artificially. The production of flavones is mainly related to the fermentation culture of* Phellinus*, which made the optimization of culture conditions an important problem. Some researches were made to optimize the fermentation culture conditions, such as the method of response surface methodology, which claimed the optimal flavones production was 1532.83 *μ*g/mL. In order to further optimize the fermentation culture conditions for flavones, in this work a hybrid intelligent algorithm with genetic algorithm and BP neural network is proposed. Our method has the intelligent learning ability and can overcome the limitation of large-scale biotic experiments. Through simulations, the optimal culture conditions are obtained and the flavones production is increased to 2200 *μ*g/mL.

## 1. Introduction

*Phellinus* is an ancient Chinese medicine, which has high medicinal value. Resent research confirmed that flavones, the secondary metabolites of* Phellinus*, can improve human immune system, reduce the side effects of anticancer agents, and relieve the reaction of patients to radiotherapy or chemotherapy [1]. In addition, flavones have positive effect on irregular menstruation and other gynecological diseases of female. There are large demands on the production of flavones, but the natural* Phellinus* is very rare. The artificial culture of* Phellinus* is hard to implement, because of the lack of culture technology and the long growth cycle of* Phellinus*. In 2008, Zeng et al. introduced a breeding method of* Phellinus* by protoplast fusion [2]. Considering the limited production of* Phellinus*, it is necessary to develop the extraction process of flavones from* Phellinus*. Fermentation is usually used to produce the secondary metabolites of fungus, such as ethanol extracts of* Phellinus baumii* [3]. It should be noticed that different fermentation methods can generate secondary metabolites with different biological activities. Currently, the production of flavones is mainly based on the fermentation culture of* Phellinus*.

In order to increase the production of* Phellinus*, the fermentation culture conditions are considered carefully, such as the fermentation temperature, the PH value, the rotation speed of the centrifuge, the inoculation volume, and the seed age. In the meantime, the composition of medium should also be taken into consideration. The multiple variables of fermentation conditions and culture mediums make the optimization by biotic experiments a hard problem. Zhu et al. took the fermentation temperature, the inoculum size, the rotation speed of the centrifuge, and bottling capacity as the independent variables and the fermentation yield as a dependent variable [4]. The quadratic regression orthogonal rotating combination design method was used to build the model for* Phellinus linteus* fermentation process, and the following optimal fermentation conditions of* Phellinus linteus* were obtained: the bottling volume is 120 mL, the inoculum size is 17 mL, the temperature is 26°C, and the rotation speed of the centrifuge is 135 r/min. At this time, the theoretical extreme value of fermentation mycelium production was 24.51 mg/mL. Other researches were focused on the fermentation parameters, such as the dosage of carbon source and nitrogen source. In 2010, Zhu et al. used response surface methodology and gave out the optimum liquid fermentation conditions as follows: the concentration of corn starch is 0.5%, the concentration of yeast extract is 2%, the concentration of VB_1_ is 0.1%, the period of fermentation is 6 days, and the production of mycelium (dry weight) of* P. linteus* is 18.43 g/L [5, 6]. It should be noticed that these researches were based on single-factor experiments, which made the outcomes rely on some presented parameters. Some machine learning strategies [7, 8] have been applied in solving problem with multiple variables and rely on fewer biotic experiments [9, 10].

In this work, we focus on the optimization of fermentation conditions, which include the concentrations of glucose, maltose, mannitol, corn powder, yeast extract, copper sulfate, sodium chloride, ferrous sulfate, and vitamin B_1_. A hybrid algorithm combined by genetic algorithm (GA) and artificial neural network (ANN) is introduced in this paper. This new algorithm has the intelligent learning ability and can overcome the limitation of large-scale biotic experiments. Through simulations, the optimal culture conditions are obtained and the flavones production is increased to 2200 *μ*g/mL.

## 2. Method

In this section, our method for optimizing the fermentation condition by BP neural network and genetic algorithm is introduced.

### 2.1. BP Neural Network

The back propagation (BP) neural network proposed in [11] is a kind of former multiway propagation network, with an input layer, an intermediate layer (hidden layer), and an output layer. The model is now known as one of the most widely applied neural network models in practice. Any neuron from the input layer has a connection to every neuron in the hidden layer, while any neuron in the hidden layer connects with every output neuron. There is no connection between each pair of neurons in the same layer [12]. BP network can be used to learn and store the relationship between the input and output. In the learning process, back propagation is used to update the weights and threshold values of the network to achieve the minimum error sum of square [13]. When a pair of the learning samples are input into the network, the neuron activation values are regulated from the output layer to the input layer, to obtain input response in the output layer neurons. With the spread of correcting such errors inversely ongoing, correct rate of the network is input to the model in response to the rise.

BP neural network is used here as a mathematic model on fermentation condition for* Phellinus igniarius*. The proposed BP neural network contains three layers of neurons:

(i) Input layer has 9 neurons to input the values of the 9 related factors of fermentation condition: glucose, maltose, mannitol, corn powder, yeast, cupric sulfate, ferrous sulfate, sodium chloride, and vitamin B_1_.
(ii) Hidden layer with 11 neurons is used to generate the scaled estimated value of* Phellinus* yield. The hidden layer neurons should be selected as an integer between 3 and 13. Here the hidden layer neurons are decided to be 11, since the variance between the predicted value and the actual value is minimum when the number of the hidden layer neurons is 11.
(iii) Output layer with one neuron is used to calculate the production of* Phellinus igniarius*.

The input and output values are limited in the range [−1,1] by

(1) $$\begin{array}{c} y=\frac{\left(x-x_{\min}\right)}{\left(x_{\max}-x_{\min}\right)}, \end{array}$$

where *x*_max⁡_ is the maximum value of the same class in the training set of data, *x*_min⁡_ is the minimum value of the same class in the training set of data, *x* is the true value of the same class in the training set of data, and *y* is the input or output value of the network.

**Figure 1:**
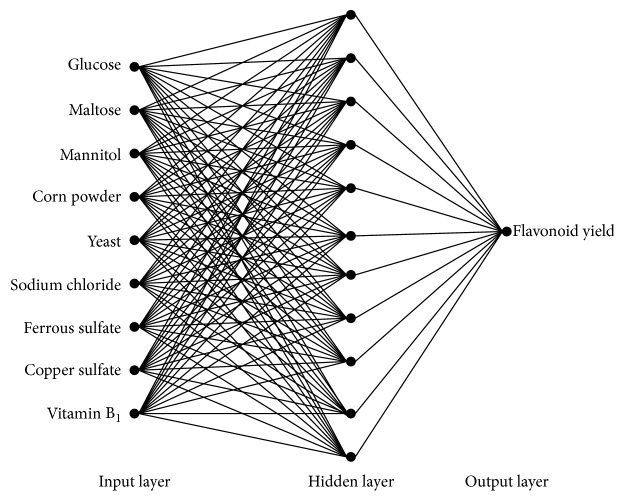
The BP neural network model.

The topological structure of the proposed BP neural network model is shown in Figure 1.

The Levenberg-Marquardt algorithm and scaled conjugate gradient method are used for training the network. Such training strategy uses both the information of the first derivative of the objective function and the information with the second derivative of the objective function, which are described as follows:

(2) $$\begin{array}{c} X^{k+1}=X^{k}+\alpha^{k}S\left(\;X^{k}\;\right), \end{array}$$

where *X* ^*k*^ is a vector composed of all the weights and thresholds in the network, *S*(*X* ^*k*^) is the search direction of the vector space composed of every component of *X*, and *α* ^*k*^ is the smallest steps using *f*(*X* ^*k*+1^) on the directory of *S*(*X* ^*k*^).

In order to establish a training set, more than 5000 experiments were completed as follows: inoculating the* Phellinus* strains on PDA slant medium and cultivating it for 7 days under the temperature of 28°C; loading 200 mL PDA liquid medium in 500 mL flask, keeping the temperature at 28°C, the speed of the centrifuge at 150 rpm, and the PH nature, and cultivating it for 7 days after inoculating; cultivating the 250 mL shake flasks for 7 days after inoculating, where the inoculation of seed liquid is 10%, the capacity of medium is 100 mL, the temperature is 28°C, and the speed is 150 rpm. Data of 25 experimental fermentation conditions with optimal production of* Phellinus igniarius* are selected from the above experiments as training set. The data of 25 experimental conditions is shown in Table 1, in which “Glu” stands for glucose, “Mal” for maltose, “Mann” for mannitol, “CP” for corn powder, “CS” for cupric sulfate, “SC” for sodium chloride, “FS” for ferrous sulfate, and “TF” for total flavonoids.

**Figure 2:**
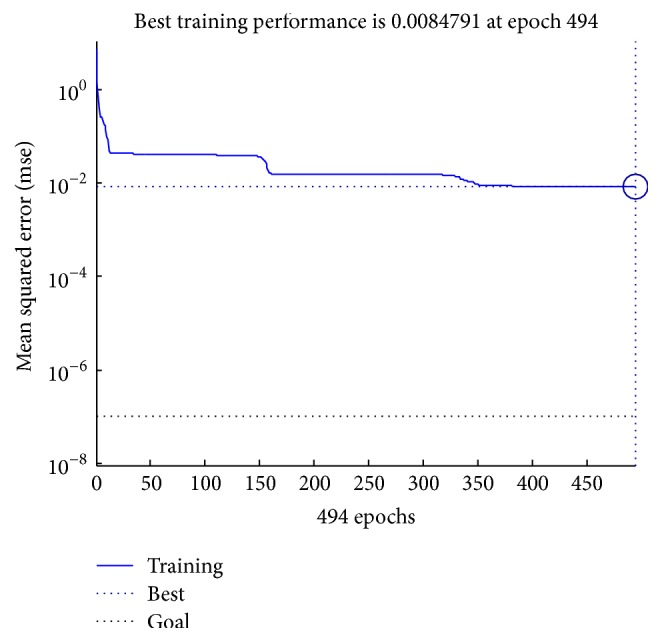
Convergence property of neural network.

Three parameters are used to measure the accuracy and speed of the network. Specifically, obtained target error (MSE) is 0.018999, operation time is 3 seconds, and the number of iterations is 577 times. Convergence property of the neural network is shown in Figure 2.

**Figure 3:**
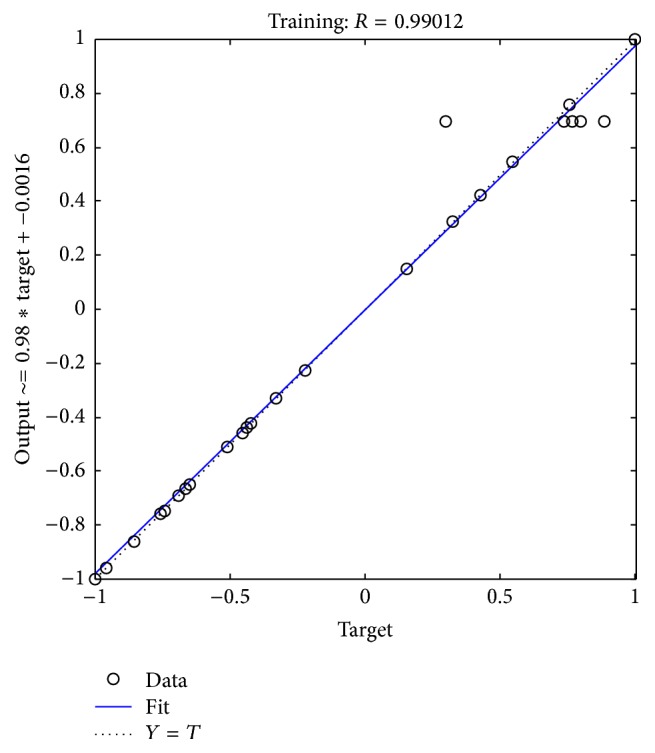
The coverage of model.

As shown in Figure 2, it is easy to find that the convergence is rapid, and the neural network converges slowly when it is closed to the target solution and finally converges to a best value. The coverage of model is shown in Figure 3.

### 2.2. Genetic Algorithm for Optimization of Fermentation Condition

In this subsection, genetic algorithm (GA) is used to optimize the fermentation condition.

GA is known as an intelligent algorithm inspired by the natural selection and genetic mechanism [14]. Similar to the basic laws of nature evolution, “survival of the fittest” is the core mechanism of the genetic algorithm; meanwhile, “reproduce,” “crossover,” and “mutation” operators are used in GA. The process has the following steps [15].

**Step 1 .**

Encode the individuals; since there are 9 variables in consideration, an individual should contain 9 bases. For example, encode [glucose, maltose, mannitol, corn powder, yeast, copper sulfate, sodium chloride, ferrous sulfate, vitamin B_1_] to [*g*_1_, *g*_2_, *g*_3_, *g*_4_, *g*_5_, *g*_6_, *g*_7_, *g*_8_, *g*_9_], where *g*_1_ ∈ [0,40], *g*_2_ ∈ [0,40], *g*_3_ ∈ [0,40], *g*_4_ ∈ [0,100], *g*_5_ ∈ [0,100], *g*_6_ ∈ [0,0.5], *g*_7_ ∈ [0,10], *g*_8_ ∈ [0,0.5], *g*_9_ ∈ [0,0.1], and the unit in use is g/L.

**Step 2 .**

Select “good” individuals according to a fitness function.

**Step 3 .**

Remove individuals with low fitness.

**Step 4 .**

Perform crossover and mutation to generate new individuals.

**Step 5 .**

Generate a new generation for evaluation by fitness function, and go to Step 2.

The process can be repeated until the halting condition is matched.

In iterations analysis, the numbers of iterations are set to be 100, 150, 200, and 500, respectively. The range of crossover probability is from 0.6 to 1, and mutation probability is from 0.01 to 0.1. The size of the population is set to be 300 and chromosome size is 9 for factors glucose, maltose, mannitol, corn pulp powder, yeast, sodium chloride, ferrous sulfate, copper sulfate, and vitamin B_1_. The encoding strategy of chromosome is floating-point (real) coding, where crossover and mutation are directly on the real operation. The concentration ranges of the factors are given as follows: glucose: 0–40 g/L, maltose: 0–40 g/L, mannitol: 0–40 g/L, corn pulp powder: 0–100 g/L, yeast: 0–100 g/L, copper sulfate: 0–0.5 g/L, sodium chloride: 0–10 g/L, ferrous sulfate: 0–0.5 g/L, and vitamin B_1_: 0–0.1 g/L.

In our method, the BP neural network obtained in Section 2.1 is used as fitness function to select good individuals. Selection operator is roulette selection method, roulette wheel selection, also known as proportional selection operator. The basic idea is that the probability of each individual selected is proportional to its fitness value. Assuming that group size is *N*, *x*_*i*_ is an individual, the fitness of *x*_*i*_ is *f*(*x*_*i*_), and the selection probability of *x*_*i*_ is

(3) $$\begin{array}{c} P\left(\;x_{i}\;\right)=\frac{f\left(x_{i}\right)}{\sum_{j-1}^{N}f\left(x_{j}\right)}. \end{array}$$

## 3. Results

Since GA starts with certain randomly selected individuals, we perform 100 data experiments. In Table 2, 10 optimized fermentation conditions for* Phellinus igniarius* are shown, where

- the average of glucose is 15.1 g/mL;
- the average of maltose is 15.264 g/mL;
- the average of mannitol is 29.83 g/mL;
- the average of corn pulp powder is 5.23 g/mL;
- the average of yeast is 5.14 g/mL;
- the average of copper sulfate is 0.18 g/mL;
- the average of sodium chloride is 9.89 g/mL;
- the average of ferrous sulfate is 0.3 g/mL;
- the average of vitamin B_1_ is 1.03 g/mL.

The average production of* Phellinus* is 2200 *μ*g/mL.

Our method has the intelligent learning ability (by BP neural network) and can overcome the limitation of large-scale biotic experiments. Through simulations, the optimal culture conditions are obtained and the flavones production is increased to 2200 *μ*g/mL from the known optimal result of 1532.83 *μ*g/mL in [6].

## 4. Conclusion

In this work, we focused on the optimization of fermentation conditions for* Phellinus igniarius*, including the concentration of glucose, maltose, mannitol, corn powder, yeast extract, copper sulfate, sodium chloride, ferrous sulfate, and vitamin B_1_. A hybrid algorithm of GA is proposed, where a BP neural network trained by 25 groups of data of experiments with optimal productions of* Phellinus igniarius* is used as the fitness function of GA. The simulation results show that our method has the ability to overcome the limitation of large-scale biotic experiments. The optimal culture conditions are obtained and the flavones production is increased to 2200 *μ*g/mL. Our work would also be a guide for the “Precision Medicine” with personal SNP data [16] and other tasks in bioinformatics [17, 18].

In our study, BP neural network is used, which is known as some classical neural computing models. It is of interest to use spiking neural networks computing models to do the optimization [19–22]. In the framework of membrane computing, cell-like [23] and tissue-like [24] computing models have been proved to be powerful as bioinspired computing models. What will happen if these models are used in calculating the optimized conditions? Some web servers are useful for biological data processing; see, for example, [25]. It is worthy to develop some web servers for experimental conditions optimization.

## Figures and Tables

**Table 1:** 25 optimal fermentation conditions of experiments for production of *Phellinus igniarius*.

Number	Glu	Mal	Mann	CP	Yeast	CS	SC	FS	VB_1_	TF
1	25	25	20	12.5	12.5	5	0.5	12.5	1.5	1.24*E* + 03
2	20	25	25	10	12.5	6.25	0.5	10	1.5	0.88*E* + 03
3	25	20	25	12.5	10	6.25	0.625	12.5	1.5	0.98*E* + 03
4	20	25	25	12.5	10	5	0.625	12.5	1.5	0.95*E* + 03
5	25	25	20	10	10	5	0.625	10	1.875	0.80*E* + 03
6	25	25	25	10	10	6.25	0.5	10	1.875	0.76*E* + 03
7	20	20	20	10	10	5	0.5	10	1.5	0.74*E* + 03
8	25	20	20	10	12.5	5	0.5	10	1.5	0.74*E* + 03
9	20	20	20	12.5	10	6.25	0.5	12.5	1.875	0.85*E* + 03
10	20	20	25	10	12.5	5	0.625	10	1.875	0.85*E* + 03
11	20	25	20	12.5	12.5	6.25	0.625	12.5	1.875	0.99*E* + 03
12	25	20	25	12.5	12.5	5	0.5	12.5	1.875	1.03*E* + 03
13	20	20	20	10	10	5	0.5	12.5	1.5	1.52*E* + 03
14	20	20	20	12.8	12	5	0.5	10	1.5	0.99*E* + 03
15	20	20	20	10	10	5	0.5	12.5	1.5	1.51*E* + 03
16	20	20	20	10	7.2	5	0.5	12.5	1.5	1.36*E* + 03
17	20	20	20	10	12.8	5	0.5	10	1.5	0.89*E* + 03
18	20	20	20	7.2	10	5	0.5	10	1.5	1.32*E* + 03
19	20	20	20	12	12	5	0.5	10	1.5	1.42*E* + 03
20	20	20	20	10	10	5	0.5	10	1.5	1.56*E* + 03
21	20	20	20	12	8	5	0.5	12.5	1.5	1.51*E* + 03
22	20	20	20	8	12	5	0.5	10	1.5	1.08*E* + 03
23	20	20	20	10	10	5	0.5	12.5	1.5	1.50*E* + 03
24	20	20	20	8	8	5	0.5	12.5	1.5	1.61*E* + 03
25	20	20	20	10	10	5	0.5	25	1.5	1.31*E* + 03

**Table 2:** 10 optimized fermentation conditions for *Phellinus igniarius*.

Number	Glu	Mal	Mann	CP	Yeast	CS	SC	FS	VB_1_	TF

1	15.04	15.2	29.97	5.27	5.09	0.18	0.22	9.8	1	2.22*E* + 03
2	15.13	15.27	29.85	5	5.16	0.2	0.23	9.82	1.08	2.22*E* + 03
3	15.04	15.63	29.81	5.16	5.11	0.19	0.55	9.96	1.01	2.22*E* + 03
4	15.03	15.64	29.98	6.35	5.39	0.16	0.4	9.81	1.11	2.20*E* + 03
5	15.09	15.1	29.82	5.1	5.24	0.2	0.23	9.99	1.08	2.23*E* + 03
6	15.13	15.04	29.6	5.1	5.15	0.19	0.22	9.74	1	2.21*E* + 03
7	15.01	15.13	29.81	5.14	5.06	0.16	0.22	9.88	1.01	2.23*E* + 03
8	15.01	15.2	29.46	5.06	5.09	0.2	0.27	9.94	1.1	2.22*E* + 03
9	15.02	15.39	29.99	5.02	5.1	0.2	0.29	9.99	1	2.23*E* + 03
10	15.52	15.04	29.98	5.21	5.09	0.18	0.32	9.98	1	2.22*E* + 03
